# Germination under Moderate Salinity Increases Phenolic Content and Antioxidant Activity in Rapeseed (*Brassica* *napus* var *oleifera* Del.) Sprouts

**DOI:** 10.3390/molecules22081377

**Published:** 2017-08-19

**Authors:** Beatrice Falcinelli, Valeria Sileoni, Ombretta Marconi, Giuseppe Perretti, Muriel Quinet, Stanley Lutts, Paolo Benincasa

**Affiliations:** 1 Dipartimento di Scienze Agrarie, Alimentari ed Ambientali, Università di Perugia, Borgo XX Giugno 74, 06121 Perugia, Italy; beatricefalcinelli90@gmail.com (B.F.); valeria.sileoni@unipg.it (V.S.); ombretta.marconi@unipg.it (O.M.); giuseppe.perretti@unipg.it (G.P.); 2 Groupe de Recherche en Physiologie Végétale (GRPV), Earth and Life Institute-Agronomy (ELI-A), Université catholique de Louvain, 5 Place Croix du Sud, Bte 7.07.13, 1348 Louvain-la-Neuve, Belgium; muriel.quinet@uclouvain.be (M.Q.); stanley.lutts@uclouvain.be (S.L.)

**Keywords:** phenolics, free, bound, non-flavonoids, tannins, DPPH

## Abstract

The use of sprouts in the human diet is becoming more and more widespread because they are tasty and high in bioactive compounds and antioxidants, with related health benefits. In this work, we sprouted rapeseed under increasing salinity to investigate the effect on free and bound total phenolics (TP), non-flavonoids (NF), tannins (TAN), phenolic acids (PAs), and antioxidant activity. Seeds were incubated at 0, 25, 50, 100, 200 mM NaCl until early or late sprout stage, i.e., before or after cotyledon expansion, respectively. Sprouting and increasing salinity slightly decreased the bound fractions of TP, NF, TAN, PAs, while it increased markedly the free ones and their antioxidant activity. Further increases were observed in late sprouts. Moderate salinity (25–50 mM NaCl) caused the highest relative increase in phenolic concentration while it slightly affected sprout growth. On the contrary, at higher NaCl concentrations, sprouts grew slowly (100 mM NaCl) or even died before reaching the late sprout stage (200 mM). Overall, moderate salinity was the best compromise to increase phenolic content of rapeseed sprouts. The technique may be evaluated for transfer to other species as a cheap and feasible way to increase the nutritional value of sprouts.

## 1. Introduction

Sprouted seeds of many plant species represent a kind of vegetable often produced at home. Their consumption have been increasing in recent years because they are considered a healthy food for their high content in bioactive compounds and antioxidants [1]. Many *Brassica* species are used for sprouting since they are known to contain vitamins (i.e., ascorbic acid), minerals, phenolics and glucosinolates (GLS), which may have health-promoting effects like antioxidant and anticancer activity [2,3,4]. On the other hand, most *Brassica* species, rapeseed included, may contain erucic acid (EA), which may cause heart lesions [5], and progoitrin, a glucosinolate having goitrogenic effects [6]. However, the rapeseed cultivars labelled as 00 (double zero) have close to nil EA and GLS contents and can thus be used with no restrictions for human feeding [7], while maintaining a high supply of phenolics.

Phenolic compounds are important components of many fruits and vegetables where they contribute to flavor, color, and sensory properties and may have important effects on the oxidative stability. The phenolic compounds are divided into different classes: flavonoids (i.e., anthocyanins, flavan-3-ols, flavones, flavanones, flavonols, etc) and non-flavonoids (phenolic acids, stilbens, lignans). A group of polyphenols shows tannin properties. Tannins are divided into hydrolysable, gallic acid and ellagic acid glucose esters, and condensed, non-hydrolysable oligomeric and polymeric proanthocyanidins [8]. Phenolics occur in plants as soluble (i.e., aglycones, free phenolic acids, glycosides, esters) and insoluble forms (phenolics bound covalently conjugated through ester bonds to cell wall components like cellulose, pectin and polysaccharides) [9,10]. Phenolic acids are simple monocyclic acids and include hydroxy derivatives of benzoic acid (C6–C1) and cinnamic acid (C6–C3). In grains and seeds, phenolic acids are often in the bound form and can be freed or hydrolyzed upon acid or alkaline hydrolysis, or by enzymes [11]. Free and bound phenolics have different bioavailability and different health effects. For example, dietary intake of bound forms may have a chemo-preventive activity against colon cancer, while free and soluble conjugated forms may be more rapidly absorbed during digestion and released in the body, inhibiting oxidation of low-density lipoprotein (LDL) cholesterol and liposomes [10]. The effect of sprouting on phenolic content and antioxidant activity has been studied in many species, including *Brassicaceae* species like radish and broccoli [2,12,13], kale and mustard [13], kohlrabi, red cabbage, rutabaga, turnip greens, turnips, garden cress, and white mustard [2]. Differences in phytochemical content reported in these studies certainly depended on species but also on the growth stage of sprouts (i.e., from three days to two weeks from the start of incubation). As far as rapeseed is concerned, to date, the available literature on the phenolic content and antioxidant activity of its sprouts is scarce [14,15], while the germination of this species has been studied thoroughly, with a focus on its tolerance to salt stress [16,17]. Several reasons were adduced for this tolerance [16,17], but no study investigated the effect on phytochemical composition. Indeed, secondary metabolites can be assumed to have a role in allowing the plant react to abiotic stresses and, actually, they are more represented in wild or ancient species [18]. For example, salinity causes oxidative stress and excess of reactive oxygen species (ROS) [19] and plants react to salt stress through the stimulation of phenylalanine ammonia lyase (PAL) [20], involved in the phenylpropanoid pathway, that increases the production of phenolics [21]. The relation between phenolic synthesis and salinity falls under the concept of phytoalexins and elicitors [22,23]. In particular, phytoalexins are molecules produced de novo by plants as a defensive response, and their biosynthesis is generally induced by elicitors, like salt stress. The role of salt stress on phenolic content changes in plants has been reviewed by Waśkiewicz et al. [22]. Thus, the aim of this work was to study the effect of germinating rapeseed under salinity as a tool to increase the nutritional value, in terms of phenolic content and antioxidant activity, of sprouts at two different growth stages.

## 2. Results

### 2.1. Germination and Seedling Growth

The germination and sprout growth performances of rapeseed under the different salt treatments are reported in Table 1.

Salinity had no effect on the total germination percentage (G) and the time to reach 50% of G (T50), whereas it slowed sprout growth. The delay of growth was important at high NaCl concentrations and increased further from the early to the late sprout stage. Sprouts of S200 were even not able to reach the desired late growth stage and many died.

### 2.2. Contents of Total Phenolics, Non-Flavonoids, Tannins and PAs

**Figure 1:**
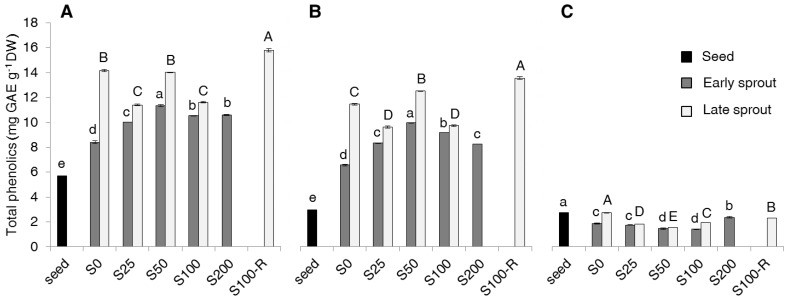
Total (**A**), free (**B**) and bound (**C**) phenolics (mg GAE g^−1^ DW) in seeds, early and late sprouts of rapeseed sprouted and grown with salt (NaCl) concentration 0, 25, 50, 100, 200 mM (S0, S25, S50, S100, S200, respectively) or sprouted with 100 mM and then grown with distilled water as recovery treatment (S100-R). Average values of duplicate determinations ± standard error are depicted. Different letters within each of the Figure 1A–C indicate statistically significant differences at *p* = 0.05 (Fisher’s least significant difference, LSD). Lower case letters are for comparison within early sprouts; upper case letters for comparison within late sprouts.

Total phenolics (TP) increased on passing from seeds to early sprouts to late sprouts (Figure 1A). With salt treatment TP increased on average, by 79% in early sprouts and by 135% in late sprouts (including S100-R). In early sprouts, TP increased with salinity up to 50 mM NaCl (+35% in S50 as compared to S0) and then decreased slightly, whereas in late sprouts it did not show a clear trend. The highest TP content was observed in S100-R. In seeds, the contents of free and bound TP fractions were almost equivalent (Figure 1B,C), while in sprouts most of the total TP was represented by the free fraction. In fact, the TP trend substantially corresponded to the trend of the free TP fraction (Figure 1B), while the bound fraction was almost unaffected, if not decreased, by either sprouting or salt treatments (Figure 1C).

**Figure 2:**
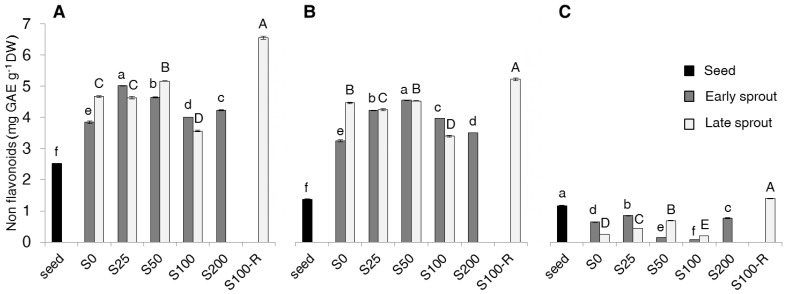
Total (**A**), free (**B**) and bound (**C**) non-flavonoid (NF) content (mg GAE g^−1^ DW) in seeds, early and late sprouts of rapeseed sprouted and grown with salt (NaCl) concentration 0, 25, 50, 100, 200 mM (S0, S25, S50, S100, S200, respectively) or sprouted with 100 mM and then grown with distilled water as recovery treatment (S100-R). Average values of duplicate determinations ± standard error are depicted. Different letters within each graph indicate statistically significant differences at *p* = 0.05 (Fisher’s LSD). Lower case letters are for comparison within early sprouts; upper case letters for comparison within late sprouts.

Around half of TP were flavonoids and half non-flavonoids both in seeds and sprouts (data not shown). Since the two fractions (flavonoids and non-flavonoids) are complementary, only non-flavonoids will be described in detail (Figure 2).

Total non-flavonoids (total-NF) (Figure 2A) were affected by either sprouting and salt treatments, showing and increase compared to seeds. In particular, total-NF in early sprouts showed the maximum increase in S25 (+30% as compared to S0) and then decreased at higher salt concentrations, while in late sprouts the effect was variable and unremarkable. As for TP, the proportion of free-NF (Figure 2B) in total NF increased dramatically passing from seeds to sprouts, while the content of bound-NF (Figure 2C) was generally very low and fluctuating among salt treatments of sprouts. The highest content of free-NF was recorded in S50 for early sprouts and in S100-R for late sprouts, with the latter showing the greatest value among all samples.

**Figure 3:**
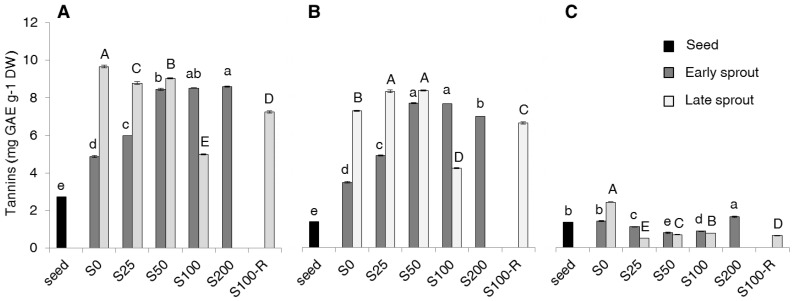
Total (**A**), free (**B**) and bound (**C**) tannin (TAN) content (mg GAE g^−1^ DW) in seeds, early and late sprouts of rapeseed sprouted and grown with salt (NaCl) concentration 0, 25, 50, 100, 200 mM (S0, S25, S50, S100, S200, respectively) or sprouted with 100 mM and then grown with distilled water as recovery treatment (S100-R). Average values of duplicate determinations ± standard error are depicted. Different letters within each graph indicate statistically significant differences at *p* = 0.05 (Fisher’s LSD). Lower case letters are for comparison within early sprouts; upper case letters for comparison within late sprouts.

The proportion of TP represented by tannic forms (TAN) compared to that of non-tannic forms was generally higher in sprouts: in fact, TAN was 47% of TP in seeds, 72% in early sprouts and 59% in late sprouts, on average over salt treatments. For this reason, TAN are described in detail (Figure 3).

Total-TAN also increased compared to seeds. In particular, in early sprouts they increased with salt concentration until S50 and kept so high in S100 and S200 (Figure 3A). Compared to early sprouts, total-TAN in late sprouts doubled in S0, increased markedly in S25 and slightly also in S50, while they showed a decrease in S100. As for TP and NF, the free-TAN (Figure 3B) represented the greatest fraction of the total-TAN and actually characterized the trend of total-TAN, while the bound-TAN fraction (Figure 3C) was very small and slightly affected by sprouting and salinity.

**Figure 4:**
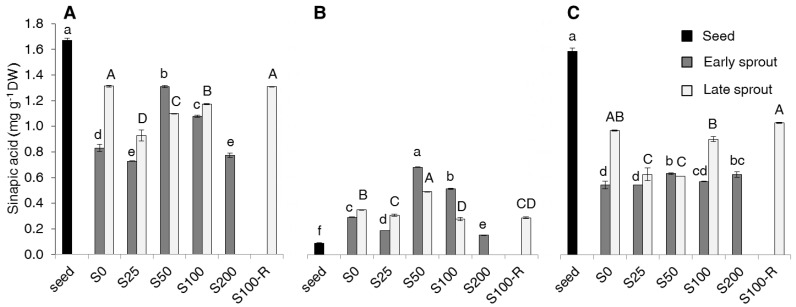
Total (**A**), free (**B**) and bound (**C**) sinapic acid (SA) content (mg g^−1^ DW) in seeds, early and late sprouts of rapeseed sprouted and grown with salt (NaCl) concentration 0, 25, 50, 100, 200 mM (S0, S25, S50, S100, S200, respectively) or sprouted with 100 mM and then grown with distilled water as recovery treatment (S100-R). Average values of duplicate determinations ± standard error are depicted. Different letters within each graph indicate statistically significant differences at *p* = 0.05 (Fisher’s LSD). Lower case letters are for comparison within early sprouts; upper case letters for comparison within late sprouts.

**Figure 5:**
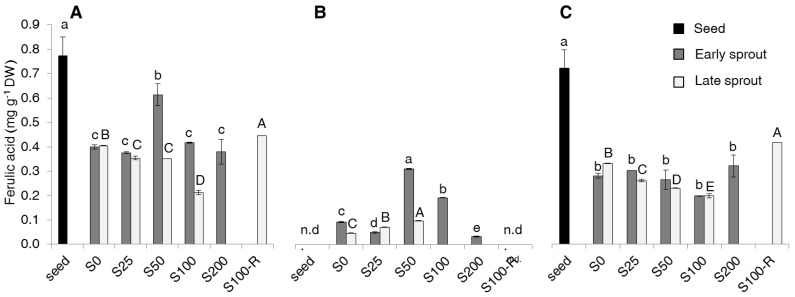
Total (**A**), free (**B**) and bound (**C**) ferulic acid (FA) content (mg g^−1^ DW) in seeds, early and late sprouts of rapeseed sprouted and grown with salt (NaCl) concentration 0, 25, 50, 100, 200 mM (S0, S25, S50, S100, S200, respectively) or sprouted with 100 mM and then grown with distilled water as recovery treatment (S100-R). Average values of duplicate determinations ± standard error are depicted. n.d., not detectable. Different letters within each graph indicate statistically significant differences at *p* = 0.05 (Fisher’s LSD). Lower case letters are for comparison within early sprouts; upper case letters for comparison within late sprouts.

As far as the phenolic acids (PAs) are concerned, of the five PAs considered, only two, sinapic acid (SA) and ferulic acid (FA) were detected (Figure 4 and Figure 5), while the other three were not detectable (data not shown).

SA was the most represented, almost double the amount of FA. For both PAs, the highest content was found in seeds in the bound forms (Figure 4C and Figure 5C). Sprouting decreased the total content (Figure 4A and Figure 5A), but increased the free forms of both PAs (Figure 4B and Figure 5B). Salinity did not give a clear effect; the only noticeable evidence was the higher content of both acids in early sprouts of S50. 

### 2.3. DPPH Scavenging Activity of Free and Bound Phenolic Fraction 

**Figure 6:**
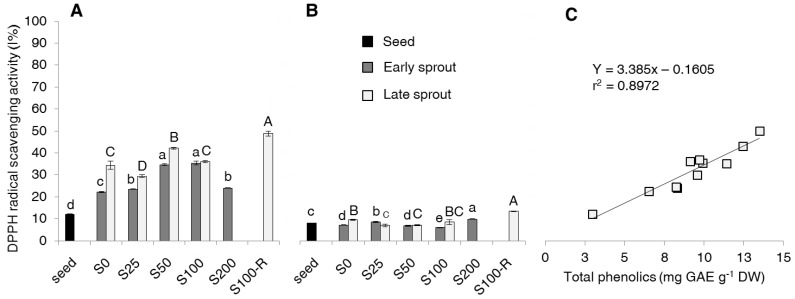
2,2-Diphenyl-1-picrylhydrazyl (DPPH) radical scavenging activity (% inhibition of DPPH radical) of (**A**) free and (**B**) bound forms of phenolics, and (**C**) correlation between DPPH radical scavenging activity and content of the free form in seeds, early and late sprouts of rapeseed sprouted and grown with salt (NaCl) concentration 0, 25, 50, 100, 200 mM (S0, S25, S50, S100, S200, respectively) or sprouted with 100 mM and then grown with distilled water as recovery treatment (S100-R). Average values of duplicate determinations ± standard error are depicted. Different letters within each graph indicate statistically significant differences at *p* = 0.05 (Fisher’s LSD). Lower case letters are for comparison within early sprouts; upper case letters for comparison within late sprouts.

The 2,2-diphenyl-1-picrylhydrazyl (DPPH) radical scavenging activity of the free fraction was much higher than that recorded for the bound fraction either in seeds or in early or late sprouts (Figure 6A). The DPPH radical scavenging of the free forms increased passing from seeds to early sprouts and then to late sprouts. Salinity caused appreciably higher DPPH radical scavenging of the free forms in S50 and S100 (as compared to the other treatments) of both early and late sprouts. The DPPH radical scavenging of the bound forms was generally not affected by either sprouting or salinity (Figure 6B). A highly significant positive linear correlation was found between free-TP and DPPH radical scavenging activity of the free fraction (Figure 6C).

## 3. Discussion

The lack of effect of salinity on the germination performance (both G and T50) up to 200 mM NaCl was expected for the rapeseed cultivar used in this experiment, based on previous findings by Pace et al. [16]. Similarly, the greater salt-sensitivity during the sprout growth confirms findings by Benincasa et al. [17], who ascertained that a salt tolerance during germination does not necessarily imply salt tolerance during seedling growth.

Results indicate that sprouting and salinity increased total phenolics in rapeseed (Figure 1). The increase with sprouting is in agreement with results from the only previous experiment on rapeseed sprouts (7-day-old), by Zieliński at al. [15]. Some evidence is also reported for other *Brassica* species [2,12,13]. Our data are in line with results by Pajak et al. [12], who reported an increase of total phenolics expressed on a dry weight basis passing from seeds to 5-day old sprouts of radish and broccoli. Data by Baenas et al. [2] are not easy to compare because these authors expressed the phenolic content on a fresh weight basis. The effect of expressing results on fresh or dry weight basis is clear when referring to Cevallos-Casals and Cisneros-Zevallos [13], who reported both. We chose to consider the dry weight in order to compare seeds and sprouts at different stages and salt treatments, which had a very different dry weight content (from 89.5% in seeds, data not shown, to about 8.0% in late sprouts of S0, as it can be calculated from data in Table 1). As far as salinity is concerned, experiments on radish [24] and broccoli [25] showed a decrease of total phenolics in sprouts obtained at increasing salinity, but again these data were expressed on a fresh weight basis. We demonstrate here that, comparing plant material on a dry weight basis, salinity increased the total phenolic content, in particular in early sprouts obtained under moderate salt concentrations (25 to 50 mM). It is worth noticing that the lower water content of sprouts obtained under salinity implies, for a given daily consumption of fresh sprouts, a much higher intake of phenols.

The increase of total phenolics with germination and salinity would respond to the need of seedlings to face adverse conditions occurring during germination, as hypothesized by Cevallos-Casals and Cisneros-Zevallos [13], or to contrast the oxidative stress caused by salinity [19,20,21]. The effect of salinity is strictly dependent on genotype sensitivity [22]. Although the initial effects of salinity are similar any genotype, in the long term physiological changes occur in salt-sentitive genotypes, which start to accumulate ions (Na^+^ and Cl^−^) more quickly than salt-tolerant ones, progressively leading to death [22]. Among rapeseed genotypes, Pace et al. [16] found the cultivar (cv.) Exagone as salt-tolerant for germination, but Benincasa et al [17] then found this cultivar was quite sensitive to salt-stress during seedling growth. This experiment confirms that sprouts were quite sensitive to salt stress. Thus, the effect of salinity as an elicitor was important in S25 and S50 while the toxic effect became prevalent in S100 and lead to death in S200. The highest total phenolic content observed in late sprouts of the recovery treatment (S100-R) is hard to discuss in lack of an analogous treatment in the literature concerning rapeseed sprouts. However, Panda and Khan [26], in 15-day old plantlets of *Vigna radiata*, observed that after removing a short-term salt stress, the concentration of Na^+^ in all plant-tissues remained high, which might explain the need for the plant to maintain a high phenolic content to contrast the induction of ROS production caused by Na^+^.

It is important to pinpoint that our total phenolics include both free and bound forms in agreement with Ti et al. [27], while most of literature intends total phenolics as the sole fraction extracted with organic solvent (i.e., the free one) [9,10]. We chose to measure both free and bound fractions because we had to compare sprouts, where the free form is prevalent, with seeds, where the bound form is half of the total phenolics and thus could not be neglected (Figure 1). The much higher increase of free phenolics compared to the decrease of bound phenolics with sprouting can be explained with additional synthesis of phenolics in response to germination, as already hypothesized by Ti et al. [27] for rice sprouts. Since total phenolics were about half flavonoids and half non-flavonoids (Figure 2), what discussed above for total phenolics stands for both these two fractions. 

With regard to tannins (Figure 3), although they are often considered as antinutrients, many studies have reported their antioxidant activity and their beneficial effect for human health, like protection against stomach and duodenal tumours, and anti-diarrhoea, anti-inflammatory and antiseptic properties [8]. Scarce information is available on the effect of sprouting and salinity in *Brassica* species. In other plant families, e.g., in many legumes, tannins are reported to decrease during germination [28,29,30,31]. However, in those experiments sprouts were obtained in the dark and harvested very early (less than 2 days after sowing), while our sprouts were grown with 200 µmol photons m^−2^s^−1^ until 4 to 11 days after sowing. Actually, plants may increase tannin production when exposed to radiation [32]. The effect of salinity on tannin content is not univocal in the literature. Reinoso et al. [33] reported that condensed tannins are accumulated by plants under different stresses, since they might be involved in the scavenging of reactive oxygen species (ROS). By contrast, Odjegba and Alokolaro [34] found that salinity decreased tannin content in leaves of *Acalypha wilkesiana*. Since bound tannins did not change markedly with germination and salinity, we may draw that free tannins were synthesized ex-novo, as discussed previously for total phenolics. 

The sinapic and ferulic acid levels recorded in our seeds (Figure 4 and Figure 5) were also found in rapeseed by Szydzowska-Czerniak et al. [35]. The sinapic acid confirmed to be the most abundant, as in other *Brassica* species [2,12]. We did not find gallic, caffeic and *p*-coumaric acids, which were detected in rapeseed seeds only by Szydzowska-Czerniak et al. [35]. The decrease of both total sinapic and ferulic acid content with sprouting in spite of the above said increase of non-flavonoids suggests that non-flavonoids produced during germination are other than PAs. No literature is available for PAs in rapeseed sprouts to make a comparison. Pajak et al. [12], working with either radish and broccoli, did not observe substantial variations in both free and bound forms of sinapic acid, while the contents of other PAs, including ferulic acid, were always very little both as free and bound forms. The effect of salinity on PAs was not clear and not so relevant and, again, no reference is available on this subject for sprouts of rapeseed and other *Brassica* species. 

In this study, antioxidant activity was measured as the capability of extracts to scavenge the DPPH radical (Figure 6). Only a couple of reference is available in the literature for antioxidant activity of rapeseed sprouts but with another antioxidant assay based on 2,2′-azinobis (3-ethylbenzothiazoline-6-sulfonic acid) diammonium salt (ABTS) radical scavenging activity, expressed as trolox equivalent (TE) [14,15]. The literature available for other *Brassica* species refers to the antioxidant activity of the sole free fraction. The antioxidant activity of our free fraction is in line with results by Pająk et al. [12] in radish and broccoli, and of Cevallos-Casals and Cisneros-Zevallos [13] in mustard, radish, broccoli and kale sprouts. The higher antioxidant activity of free phenolics compared to that showed by bound phenolics was also observed by Ti et al. [27] in rice seedlings using ferric reducing antioxidant power (FRAP) and oxygen radical absorbance capacity (ORAC) assays. With regard to salinity, the only references available for *Brassica* species [24,25] used different essays and expressed the antioxidant activity on a fresh weight basis. Looking at other plant families, Lim et al. [21] reported similar effects of salinity in buckwheat sprouts. Combining the results in Figure 6 with those in previous figures it can be deduced that the increase of antioxidant activity observed with both sprouting and salinity is mainly ascribable to free phenolics.

## 4. Materials and Methods 

### 4.1. Plant Material and Experimental Design

Seeds of rapeseed (*Brassica napus* var *oleifera* Del.) cv. Exagone, which had been found to be tolerant to salt stress [16], were used in this study. The seeds belonged to the same lot used for previous studies [16,17] and had been provided directly by Monsanto Italia (Milano, Italy) in 2010. They had been stored under vacuum at low temperature and a preliminary test revealed that their germination performance was still very good.

Seeds were incubated in plastic trays containing solutions with 0, 25, 50, 100, 200 mM NaCl (treatments S0, S25, S50, S100, S200, respectively) according to a completely randomized block design with four replicates (trays). Each tray contained 5 g of seeds, corresponding to over 1000 seeds. Sprouting conditions were chosen in analogy with the method adopted in Benincasa et al. [18]. In detail, seeds were positioned on filter paper laid over glass balls immersed in the solution contained into the trays, in order to guarantee constant water availability and prevent anoxia. Distilled water was periodically added to trays to restore initial tray weight, assuming that weight loss was mainly due to water evaporation [18], so approximately keeping the initial NaCl concentration of each treatment [17]. The trays were kept in a growth chamber at 18 °C in the dark. After germination, the trays were placed at a light/dark regime of 16/8 h with light intensity of 200 µmol photons m^−2^s^−1^. Rapeseed sprouts were collected at two different stages of development labelled as “early sprout”, upon the elongation of shoot and before the expansion of cotyledons, and “late sprout”, after the complete development of cotyledons. Early and late sprouts in S0 were collected 4 and 7 days after sowing (DAS). Since increasing salinity slowed seedling growth, early and late sprouts of the other salt treatments were collected when they reached the growth stage as in the unsalted control: i.e., 4 DAS for early sprouts and 8 DAS for late sprouts in S25, 5 and 9 DAS in S50, 6 and 11 DAS in S100, 8 DAS for early sprouts in S200. Late sprouts from S200 were not collected since no individual was able to reach that stage and many died. A recovery treatment (S100-R), obtained by transferring early sprouts from 100 mM NaCl to 0 mM NaCl until the late sprouts stage (9 DAS), was included.

Replicates of each treatment were re-grouped two by two in order to get two samples per treatment for the chemical analysis. Samples were stored at −20 °C until needed. One extract was obtained from each sample and each extract was measured in duplicate.

Fresh and dry weights of either early or late sprouts were measured on 10 individuals per replicate. The dry weight was measured following the Association of Official Analytical Chemists’ (AOAC) methods 925.10 [36].

The germination percentage (G) and time to reach 50% germination (T50) were measured by running a separate germination test with 4 replicates (Petri dishes) of 50 seeds each per treatment.

### 4.2. Chemicals and Apparatus

Sodium lauryl sulphate (SLS) and acetonitrile were purchased from Carlo Erba (Milan, Italy). Methanol, sodium hydroxide, sodium sulfate anhydrous, hydrochloric acid (37% *w*/*v*), sodium carbonate, acetic acid, ferulic acid, sinapic acid, gallic acid, caffeic acid, *p*-coumaric acid, and 2,2-diphenyl-1-picrylhydrazyl (DPPH) were purchased from Sigma Aldrich (St. Louis, MO, USA). All other chemicals used were of an analytical grade. A Cary 100 UV-Vis spectrophometer (Agilent, Santa Clara, CA, USA) was used for spectrophotometric analysis. 

### 4.3. Extraction of Free and Bound Phenolic Fractions

The extraction of free and bound phenolics fractions was achieved following the method of Benincasa et al. [18]. Seeds or sprouts (2 g) were used for the extraction. Extracts were dried and stored at −20 °C until chemical analysis. Before chemical measurements, extracts were dissolved in 1 mL of methanol for the bound phenolic fraction and in 2 mL for the free fraction. After appropriate dilution (1:10), aliquots of extracts for the free and bound fractions were used for determination of TP, NF, TAN, PAs, and DPPH assay.

### 4.4. Total Phenolics

The total phenolic content was measured following the method of Singleton and Rossi [37], with phosphomolybdic—phosphotungstic acid reagent (Folin-Ciocalteu reagent). Aliquots (200 µL) of extracts for the free and bound fractions were mixed with 2 mL of water, 10 mL of Folin-Ciocalteu reagent and 8 mL of sodium carbonate 7.5% (Na_2_CO_3_). After two hours the absorbance was read at 765 nm. Gallic acid was used as a standard and results were expressed as milligrams of gallic acid equivalent per gram of sample dry weight (mg GAE g^−1^ DW). Total phenolic content was expressed as the sum of free and bound fractions.

### 4.5. Total Non-Flavonoid and Tannic Content

For non-flavonoids, aliquots of the diluted extracts for the free and bound fractions (1 mL) were mixed with hydrochloric acid 1:4 (*v*/*v*) (1 mL) and formaldehyde (0.5 mL). Non-flavonoid extracts were kept at room temperature for 24 h. For the non-tannic fraction, aliquots of diluted extracts (1 mL) were mixed with methylcellulose (0.2 mL), ammonium sulfate (0.4 mL) and distilled water (0.4 mL) and then centrifuged (3000 g) for 15 min.

The concentration of non-tannic phenols was evaluated in the supernatant after selective precipitation with methylcellulose [38], whereas the amount of the non-flavonoid fraction was evaluated after precipitation of the flavonoid fraction with formaldehyde [39]. Tannic phenols was obtained by subtraction of the phenolic content. Aliquots (0.4 mL) of the surnatant for either non-flavonoids or non-tannins were mixed with 2 mL of Folin-Ciocalteu reagent (1:10) and sodium carbonate (Na_2_CO_3_) 7.5% (1.6 mL). After 2 h at room temperature, absorbance was read at 765 nm. Gallic acid was used as a standard and results were expressed as milligrams of gallic acid equivalent per gram of sample dry weight (mg GAE g^−1^ DW). Either total non-flavonoids or total tannins were expressed as the sum of free and bound fractions. 

### 4.6. Analysis of Phenolic Acids Contents

The determination of PAs was achieved according to the method of Floridi et al. [40], which is based on high performance liquid chromatography (HPLC) coupled with a CoulArray detector for an electro-chemical detection (HPLC-ECD) using a solvent gradient. Mobile phase A was 0.05 M KH_2_PO_4_ and 0.05 µM SLS, and mobile phase B was phase A/CH_3_OH/CH_3_CN, 30:20:50 *v*/*v*/*v*, 0.05 µM SLS. The gradient cycle was as follows: 85% of phase A at the starting point followed by a decrease to 80% in 5 min, held for 30 min, increased to 85% in 1 min, held for 16 min, decreased to 50% in 20 min, held for 1 min, decreased to 0% in 1 min, held for 7 min, brought back to the initial conditions in 2 min and finally held to 85% of phase A for 5 min. A flow rate was also applied: 0.9 mL/min at the starting point, held for 8 min, decreased to 0.4 mL/min in 1 min, increased to 0.9 mL/min for 16 min, held for 1.5 min, decreased to 0.5 mL/min in 13 min, increased to 0.9 mL/min in 4.5 min and constant until the end of the run.

The following equipment was utilized for the HPLC analysis: two Jasco PU-1580 pumps (Jasco Inc., Easton, MD, USA) connected to a gradient solvent system, a Basic Marathon Autosampler (Spark Holland, Emmen, The Netherlands), an Inertsil ODS-3V C18 column (GL Sciences, Tokyo, Japan, 250 mm × 4.6 mm i.d., 5 µm), a CoulArray (ESA, Inc., Chelmsford, MA, USA) detector, consisting of two cell packs in series, each pack containing four porous graphite working electrode channels with associated palladium reference electrode and platinum counter electrode, and CoulArray Software for Windows for acquisition and elaboration of data.

Standards of phenolic acids (sinapic acid, SA; ferulic acid, FA; gallic acid, GA; caffeic acid, CA; *p*-coumaric acid, *p*-CA) were prepared as stock solution at 1 mg mL^−1^ in methanol and stored at −20 °C. The 5 phenolic compound standard solutions were prepared by combining and diluting the individual stock standard solutions to obtain the desired concentrations in the range of 1–20 µg mL^−1^ for each acid. Calibration solutions were obtained by diluting the working standard mixtures, and calibration curves were obtained for each phenolic acid (*r* = 0.9909 for SA; *r* = 0.9906 for FA; *r* = 0.9938 for GA; *r* = 0.9959 for CA; *r* = 0.9865 for *p*-CA). The limit of detection (LOD) was 1.006 µg mL^−1^ for SA, 1.003 µg mL^−1^ for FA, 0.58 µg mL^−1^ for GA, 0.08 for CA and 0.13 µg mL^−1^ for *p*-CA. The contents of phenolic acids were measured in the extracts for either the free or the bound fractions and expressed as milligrams per gram of sample dry weight (mg g^−1^ DW). The total PAs contents were expressed as the sum of the free and bound fractions. 

### 4.7. DPPH Assay

The scavenging activity of the DPPH radical was measured following the method of Nencini et al. [41]. Methanol solution of 10^−4^ M DPPH was prepared freshly and stored at 4 °C in darkness until needed. Aliquots (50 µL) of diluted extracts for the free and bound fractions, or methanol for the control, were mixed with 950 µL of DPPH and were quickly incubated at room temperature in darkness for 30 min. The decrease in absorbance of DPPH solution was evaluated at 515 nm. The capability to scavenge the DPPH radical was calculated as the percentage inhibition of DPPH radical using the following equation:

(1) $$\mathrm{Inhibition}\;\mathrm{of}\;\mathrm{DPPH}\;\mathrm{radical}\;\left(\%\right)=\frac{\left(A_{0}-A\right)}{A_{0}}\;\times \;100$$

where A_0_ is the absorbance of DPPH without sample and A is the absorbance of sample with DPPH.

### 4.8. Statistical Analysis

All data were analysed by one-way ANOVA. Average values of duplicate determinations ± standard error are depicted. Means were compared by using the Fisher’s least significant difference (LSD) at *p* = 0.05. The R statistical environment was used to perform the analysis [42].

## 5. Conclusions

Our results indicate that sprouting and increasing salinity in rapeseed decreased the bound fractions of total phenolics, non-flavonoids, tannins and phenolic acids, while it increased the free phenolic fractions and their antioxidant activity. A further increase was observed passing from early sprouts to late sprouts. On the other hand, increasing salinity slowed sprout growth, especially at the highest NaCl levels, and this can partly complicate the sprouting process. Overall, the best compromise to obtain a high phenolic content and antioxidant activity was represented by moderate salt concentration (i.e., 25 or 50 mM NaCl). The technique of sprouting under moderate salinity may be evaluated for transfer to other species as a cheap and feasible way to increase sprout nutritional value.

## Figures and Tables

**Table 1:** Total germination percentage (G), time to reach 50% of G (T50), number of days after sowing (DAS) needed to reach the early sprout and late sprouts stages fixed for harvest (i.e., before and after cotyledon expansion), and correspondent individual sprout fresh (FW) and dry weights (DW) in rapeseed sprouted and grown with salt (NaCl) concentration 0, 25, 50, 100, 200 mM (S0, S25, S50, S100, S200, respectively) or sprouted with 100 mM and then grown with distilled water as recovery treatment (S100-R). Standard error in brackets.

Treatment	G (%)	T50 (d)	Early Sprouts			Late Sprouts		
			DAS	FW (mg)	DW (mg)	DAS	FW (mg)	DW (mg)
S0	99	2.5	4	28.7(3.74)	3.51(0.04)	7	37.3(1.04)	2.96(0.019)
S25	97	2.5	4	22.8(3.42)	3.60(0.16)	8	36.0(5.29)	3.62(0.091)
S50	99	2.5	5	12.3(0.67)	3.42(0.01)	9	20.4(1.84)	3.56(0.169)
S100	97	2.5	6	15.3(0.61)	3.54(0.29)	11	20.7(2.67)	3.83(0.379)
S200	96	2.5	8	13.6(0.92)	3.29(0.34)	-	-	-
S100-R	98	2.5	6	-	-	9	23.2(2.39)	3.95(0.152)
